# Applications of Big Data Analytics to Control COVID-19 Pandemic

**DOI:** 10.3390/s21072282

**Published:** 2021-03-24

**Authors:** Shikah J. Alsunaidi, Abdullah M. Almuhaideb, Nehad M. Ibrahim, Fatema S. Shaikh, Kawther S. Alqudaihi, Fahd A. Alhaidari, Irfan Ullah Khan, Nida Aslam, Mohammed S. Alshahrani

**Affiliations:** 1 Department of Computer Science, College of Computer Science and Information Technology, Imam Abdulrahman Bin Faisal University, P.O. Box 1982, Dammam 31441, Saudi Arabia; shikah.sunaidi@gmail.com (S.J.A.); nmaIbrahim@iau.edu.sa (N.M.I.); 2190500127@iau.edu.sa (K.S.A.); iurab@iau.edu.sa (I.U.K.); naslam@iau.edu.sa (N.A.); 2 Department of Networks and Communications, College of Computer Science and Information Technology, Imam Abdulrahman Bin Faisal University, P.O. Box 1982, Dammam 31441, Saudi Arabia; faalhaidari@iau.edu.sa; 3 Department of Computer Information Systems, College of Computer Science and Information Technology, Imam Abdulrahman Bin Faisal University, P.O. Box 1982, Dammam 31441, Saudi Arabia; fsshaikh@iau.edu.sa; 4 Department of Emergency Medicine, College of Medicine, Imam Abdulrahman Bin Faisal University, P.O. Box 1982, Dammam 31441, Saudi Arabia; msshahrani@iau.edu.sa

**Keywords:** artificial intelligence (AI), big data, big data analytics, 2019 novel coronavirus disease (COVID-19), healthcare

## Abstract

The COVID-19 epidemic has caused a large number of human losses and havoc in the economic, social, societal, and health systems around the world. Controlling such epidemic requires understanding its characteristics and behavior, which can be identified by collecting and analyzing the related big data. Big data analytics tools play a vital role in building knowledge required in making decisions and precautionary measures. However, due to the vast amount of data available on COVID-19 from various sources, there is a need to review the roles of big data analysis in controlling the spread of COVID-19, presenting the main challenges and directions of COVID-19 data analysis, as well as providing a framework on the related existing applications and studies to facilitate future research on COVID-19 analysis. Therefore, in this paper, we conduct a literature review to highlight the contributions of several studies in the domain of COVID-19-based big data analysis. The study presents as a taxonomy several applications used to manage and control the pandemic. Moreover, this study discusses several challenges encountered when analyzing COVID-19 data. The findings of this paper suggest valuable future directions to be considered for further research and applications.

## 1. Introduction

On 30 January 2020, the World Health Organization (WHO) declared the spread of the COVID-19 pandemic as a cause of concern and called for raising the level of health emergencies. Afterward, the government of the Kingdom of Saudi Arabia urgently took several strict measures to limit the spread of the pandemic within the regions of Saudi Arabia [1,2]. The Saudi Ministry of Health (MoH) and many other countries have implemented WHO recommendations related to the identification and isolation of suspected COVID-19 cases.

Nevertheless, the pandemic has spread dramatically, with the number of infected people over 82 million, and the number of deaths exceeding one million [3]. The rapid spread of the pandemic, with its continuous evolving patterns and the difference in its symptoms, makes it more difficult to control. Moreover, the pandemic has affected health systems and the availability of medical resources in several countries around the world, contributing to the high death rate [4].

A regular monitoring and remote detection system for individuals will assist in the fast-tracking of suspected COVID-19 cases. Moreover, using such systems will generate a huge amount of data, which will provide many opportunities for applying big data analytics tools [5] that are likely to improve the level of healthcare services. There are a large number of open-source software such as the big data components for the Apache project [6], which are designed to operate in a cloud computing and distributed environment to assist in the development of big data-based solutions. Furthermore, there are several key characteristics of big data called the Six V’s [7], namely, Value, Volume, Velocity, Variety, Veracity, and Variability. However, the original definition of the big data key characteristics considers only three Vs, namely Volume, Velocity, and Variety [8].

The big data characteristics apply to data acquired from the healthcare sector, which increases the tendency to use big data analysis tools to improve sector services and performance. There are wide applications of big data analytics in the healthcare sector, including genomics [9], drug discovery and clinical research [10], personalized healthcare [11], gynecology [12], nephrology [13], oncology [9,12], and several other applications found in the literature. However, in this paper, we present the contributions of the most important review papers found in the literature that cover the field of big data in healthcare. We also investigate the opportunities and challenges for applying big data analytics tools to COVID-19 data and provide findings and future directions at the end of the paper.

Promising wearable technology is expected to be one of the primary sources of health information, given its widespread availability and acceptance by people. Based on a survey conducted in January 2020, 88% of 4600 subjects included in the study indicated a willingness to use wearable technology to measure and track their vital signs. While 47% of chronically ill patients and 37% of non-chronically ill patients reported a willingness to blindly share their health information with healthcare research organizations. Of the same group, 59% said they would likely use artificial intelligence (AI)-based services to diagnose their health symptoms [14]. People sharing such data routinely will greatly increase the volume of data, which calls for planning to design and implement data analysis tools and models in this sector.

Several studies used big data for sentiment analysis, such as Reference [15], which linked between social media behavior and political views, opinions, and expressions. The study consisted of a representative survey conducted on 62.5% of adults from Chile and it showed the huge effect of social media on changing people’s opinions regarding political views and elections. Similarly, the authors of Reference [16] had studied how the management responding to customer satisfaction online review affects the choice of the customers for some facilities or hotels. It showed a positive correlation between the response and customer satisfaction. The authors of Reference [17] had reviewed the classification techniques, including deep and convolutional, to identify the writer from their handwriting. They discussed several challenges in identification related to language characteristics, scripts, and the lack of datasets. Also, the authors of Reference [18] had reviewed and analyzed the latest papers about big data analytics latest developments, capabilities, and profits. Their study showed that big data can support business industries in many functionalities including prediction, planning, managing, decision-making, and traceability. The limitation of their study is the data sources, which were hard to find due to privacy and conservation of the information. Moreover, the authors of Reference [19] had surveyed numerous papers about mathematical models to improve the efficiency in detecting and predicting COVID-19. Their survey suggested using artificial intelligence to detect COVID-19 cases, big data to trace cases, and nature-inspired computing (NIC) to select suitable features to increase the accuracy of detection. Some surveys studied heart-related diseases and suggested some recommendations and guidelines, such as Reference [20], to help people in understanding heart failure causes, symptoms, and the most affected group. They declared that heart failure can escalate the patient’s injuries, especially the ones with serious illnesses.

Analyzing health data in real-time with the utilization of AI techniques will have a vital role in predictive and preventive healthcare. For example, it will help predict the sites of infection and the flow of the virus. It will also help in estimating the needs of beds, healthcare specialists, and medical resources during such pandemic crises as well as in the diagnosis and characterization of the virus [21].

Several reviews in the literature have examined big data analytics in healthcare from various aspects. Table 1 summarizes a number of such studies. In this paper, we focus on identifying the applications of big data analytics for COVID-19 and the challenges that may hinder its utilization.

The rest of this paper is organized as follows. Section 2 presents the current big data analytics applications for COVID-19. Section 3 shows several tools used for big data analytics. Section 4 discusses big data analytics in the healthcare sector from different aspects and analyzes the challenges that may hinder its application, then provides our future predictions in terms of using big data in the healthcare field, in addition to several recommendations. Finally, Section 5 concludes the paper.

## 2. Applications of Data Analytics in COVID-19

**Figure 1:**
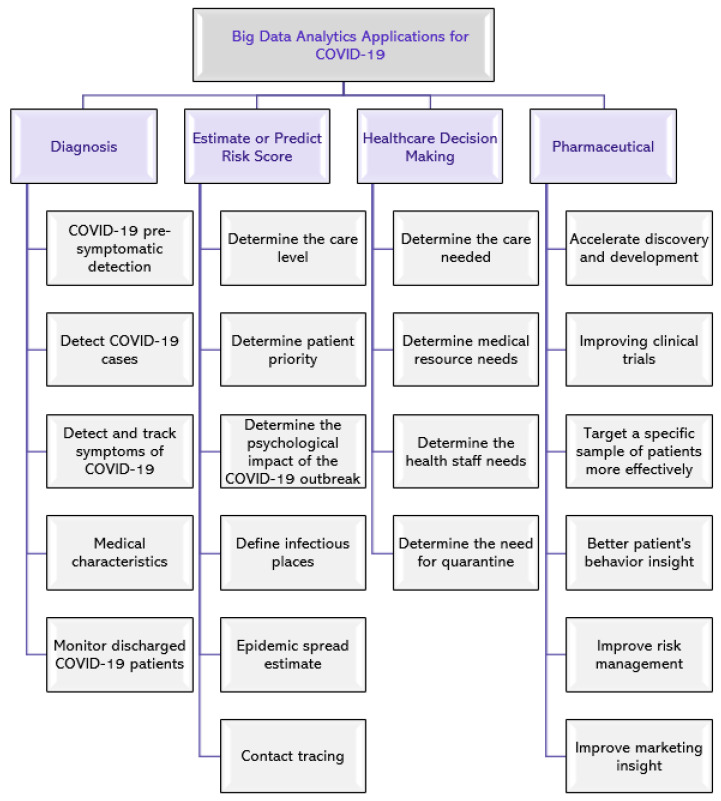
Potential application areas of big data analytics for COVID-19.

The spread of the global pandemic, COVID-19, has generated a huge and varied amount of data, which is increasing rapidly. This data can be used by applying big data analytics techniques in multiple areas, including diagnosis, estimate or predict risk score, healthcare decision-making, and pharmaceutical industry [38]. Figure 1 shows examples of potential application areas.

In the following subsections, we present several examples of COVID-19 data utilization from the literature with a primary focus on reviewing studies that have provided solutions to control the COVID-19 pandemic and fall within one of the three areas, namely (1) diagnosis (Section 2.1), (2) estimate or predict risk score (Section 2.2), and (3) healthcare decision-making (Section 2.3). We also summarize the data analysis techniques and the data type used for each study in Table 2.

### 2.1. Diagnosis

Suspected COVID-19 cases are diagnosed using the Reverse Transcription-Polymerase Chain Reaction (RT-PCR) test. This test takes around 24 h to several days, depending on the multiple conditions. Many countries experienced increased demand for diagnosing suspected COVID-19 cases, which exceeded the available local testing capacity. Therefore, several researchers have proposed alternative solutions for the COVID-19 RT-PCR diagnosis test, including the following.

The authors in Reference [39] have proposed a model to differentiate between COVID-19 and four other viral chest diseases. The model utilizes several body sensors to collect information and monitor the patient’s health condition, including temperature, blood pressure, heart rate, respiratory monitoring, glucose detection, and others. The collected data is stored on a cloud database containing AI-enabled expert systems that help diagnose symptoms of patients infected or suspected of having COVID-19 to determine the appropriate procedure to deal with them. However, it is not clear how the patient’s health information will be presented to the hospital staff. Moreover, the authors in Reference [19] had surveyed numerous papers about mathematical models to improve the efficiency in detecting and predicting COVID-19. Their survey suggested using artificial intelligence to detect COVID-19 cases, big data to trace cases, and nature-inspired computing (NIC) to select suitable features to increase the accuracy of detection.

In Reference [40], the authors provided a flexible and low-cost design of a medical device that can be used to detect and track symptoms of COVID-19. It utilizes headphones and a mobile phone to detect breathing problems. The signals are collected and saved in an audio file format through the mobile app, after which the signals are analyzed using the MATLAB program to identify the respiratory symptoms associated with COVID-19.

Researchers [41] also developed a program to remotely monitor discharged COVID-19 patients. Each patient registered to the app is provided with a pulse oximeter and thermometer to self-report daily symptoms, O2 saturation, and temperature. The abnormal vital signs and symptoms are flagged to be assessed by a group of nurses. Depending on the evaluation outcome, the patient might be readmitted to the Emergency Department (ED). The program helps reduce ED utilization and provides scalable remote monitoring capabilities when a patient is discharged from the hospital.

The authors in Reference [42] found that smartwatches could be utilized in COVID-19 pre-symptomatic detection. They analyzed the physiological and activity data collected from smartwatches of the infected COVID-19 cases. They concluded that 63% of COVID-19 cases could be detected before symptoms appear by applying a two-level warning system based on severe elevations in resting heart rate relative to individual baseline. Moreover, they found that activity tracking and health monitoring using wearable devices can help in early detection of respiratory infections.

Since the COVID-19 symptoms have not been fully identified and due to the changing nature of COVID-19, some studies have focused on identifying the medical characteristics and symptoms associated with positive COVID-19 cases. The study in Reference [43] focused on identifying the symptoms associated with the positive results of the COVID-19 examination, and it was conducted on a group of healthcare workers (HCWs). Initial screening was performed by phone, and a COVID-19 PCR test was also performed for each HCW to identify symptoms associated with each case. The study found that the most common symptoms of positive COVID-19 cases were fever, myalgia, and anosmia/ageusia, while the negative cases mostly have no symptoms, or the symptoms are limited to nasal congestion and sore throat.

The study in Reference [44] aimed to determine the clinical characteristics and outcomes of 5700 hospitalized patients with COVID-19 in the NY area. However, the study included non-critically ill patients and the follow-up time was limited.

Another study [45] proposed a website and Android app to separate a COVID-19 cough sound from other respiratory sounds with the aid of crowdsourcing data from about 7000 unique users (more than 200 of whom reported a recent positive test for COVID-19). Their proposed method employed Logistic Regression (LR), Gradient Boosting Trees, and Support Vector Machines (SVMs) classifiers to distinguish the cough sound data based on gender, age, and symptoms. Also, their classifiers distinguish the user based on other features, such as whether they are asthmatic patients, smokers, or healthy. Their app asks the user to cough from three to five times then repeat the process every two days to update the user’s health status. Their method proved that a COVID-19 cough can be distinguished from other lung diseases coughs from the sound of the cough combined with breathing sound to screen the disorder. It achieved 82% Area Under the Curve (AUC) in identifying the cases that tested positive for COVID-19. They recommended more studies in the field to specify more characteristics of a COVID-19 cough sound to make it more distinguishable from other respiratory sounds.

The authors in Reference [46] declared the importance of using complementary technologies such as on-body sensors for diagnosing and monitoring COVID-19 infections. They stated that clinical devices are more reliable and provide more functions than smartwatches since these devices are distributed in different areas of the human body to detect different body signals. A thin, soft sensor with a high-bandwidth accelerometer and a precision temperature sensor placed on the neck is very important to record respiratory activity from cough frequency, intensity, and duration to respiratory rate and effort, to high-frequency respiratory features associated with wheezing and sneezing. Also, they recommended machine learning and predictive algorithms to help to diagnose and monitor COVID-19.

In Reference [47], researchers emphasized on the importance of identifying the characteristics of COVID-19 among patients of Saudi Arabia in managing the pandemic. The study included 1519 cases where data related to their ages, genders, vital signs, public data, and clinical examinations were collected. Their test was conducted based on the quantitative RT-PCR approach, which is the protocol established by the World Health Organization. After the data was gathered, it was entered into electronic sheets with distinct data collectors, and data was analyzed with Statistical Package for Social Sciences program, version 24 (SPSS-24). The statistics manifested that the most common symptoms of COVID-19 are cough and fever, with 89.4% and 85% presence in reported positive cases, respectively. Also, it confirmed that the most infected patients’ demographics include elder males, severe cardiac condition patients, and diabetic patients.

The authors in Reference [48] had utilized machine learning techniques along with spark-based linear models, Multilayer Perceptron (MLP), and Long Short-Term Memory (LSTM) with a two-stage cascading platform to enhance the prediction accuracy in different datasets. They applied their method on two datasets for cardiac arrhythmia and resource locator, so their model performed with higher accuracy and lower computation time. Thus, the authors in Reference [49] had proposed a computer program method to aid the classification model to analyze the retinal image of diabetic retinopathy to investigate its effect among adults in causing blindness. It proved that the focused connection among layers of the convolutional network assists the accuracy of the classification result.

The retrospective, observational study in Reference [50] conducted a statistical analysis to show the cardiovascular implications of COVID-19 on the patients. The study was performed on 116 patients who tested positive for COVID-19. The data was clinically collected and tested to extract clinical symptoms and signs, chest computed tomography, treatment measures, and medical records. The statistical analysis was performed on the data to reveal similar results as those reported by Reference [47], where the common symptoms were fever and dry cough, and the elder or middle-aged males, heart injury patients, hypertension patients, and diabetics were the most infected populations.

### 2.2. Estimate or Predict Risk Score

Estimating the risk score helps in determining the care level and priority for each patient with an insight to the necessary proactive measures. In the following section, we present the studies that cover this area.

In Reference [51], the authors aimed to validate a hypothesis that COVID-19 infection could lead to serious cardiovascular diseases or maybe worse. They utilized statistical analysis by employing a multi-factorial logistic regression model to analyze COVID-19-related causes. The study was conducted on 54 patients with different ages, genders, and vital signs, where 39 were diagnosed as severe COVID-19 cases and 15 as critical COVID-19 cases. The data was collected clinically from the patients with attached vital sign measurement devices updated every four hours. Results showed that elder males, diabetic patients, and hypotension patients are more likely to develop a serious heart-related condition and need more care. Their proposed study is limited due to the small sample size, and they suggested a higher sample size to conduct a more appropriate study and verify the results.

The authors in Reference [52] are interested in developing and validating the risk score to predict adverse events among patients suspected of having COVID-19. They conducted a retrospective cohort study of adult visits to the emergency department. The study concluded that the primary outcome was death or no respiratory decompensation within 7 days. To derive the risk score, they used the Least Absolute Shrinkage and Selection (LASSO) and Logistic Regression models. They concluded that the COVID-19 Acuity Score (COVAS) can assist in decision-making to discharge patients during the COVID-19 pandemic. They also reported the derivation and validation metrics of cohorts and subgroups with pneumonia or COVID-19 diagnosis.

The authors in Reference [53] proposed an Internet of Things (IoT) based system to discover unregistered COVID-19 patients, as well as infectious places. This would help the responsible authorities to disinfect contaminated public places and quarantine the infected persons and their contacts even if they did not have any symptoms. The newly confirmed and recovered cases would be recorded in the system by the healthcare staff, while the geolocation data will be collected automatically by Global Positioning System (GPS) technology in the IoT devices. The authors discussed how their proposed system could be utilized to apply three different prediction mathematical models, namely the θ-SEIHRD model, Susceptible-Infected-Recovered (SIR) model, and Susceptible-Exposed-Infectious-Removed (SEIR) model.

Another study [54] demonstrated the possibility of transmitting the COVID-19 virus through indirect contact, like touching surfaces contaminated with the droplets of an infected person. Therefore, it was recommended that paying attention to personal hygiene and disinfection of public places could possibly reduce the incidence.

Furthermore, researchers also [55] conducted a cross-sectional study to show the impact of the COVID-19 outbreak on the psychological side. They found that fear of a COVID-19 outbreak can have significant psychological repercussions on people, which requires more attention by the relevant authorities to cope with this impact. Also, the authors in Reference [56] had proposed a model that identified the risk of getting infected by tuberculosis based on several factors related to tuberculin skin, age, and weak immune system. They stated that those factors can increase the infection from 10% to 20%.

The authors in Reference [57] provided a model that predicts the course of the outbreak to help plan an efficient method of prevention. Model stages are SIDARTHE (susceptible, infected, diagnosed, ailing, recognized, threatened, healed, and extinct). It discriminates between infected people based on whether they have been diagnosed and on the severity of their symptoms. The simulation results obtained by combining the model with the available data on the COVID-19 pandemic in Italy indicate that it is an urgent necessity.

### 2.3. Healthcare Decision-Making

During the COVID-19 pandemic, the demand for emergency departments and medical equipment such as ventilators increased. Therefore, many studies have aimed to provide monitoring tools and models that help in making several medical decisions to mitigate potential risks, and these solutions include the following.

The authors in Reference [58] designed a prediction model called Conscious-based Susceptible-Exposed-Infective-Recovered (C-SEIR) model to ensure the usefulness of the lockdown and protective countermeasures in decreasing the influence of the pandemic in Wuhan city. The proposed model consisted of two classification groups, namely the quarantined suspected infection group (P), and the quarantined diagnosed infection group (Q), along with a blue/green curve with a solid line for daily patients and dashed line for cumulative patients. It showed that the result of the prediction is a double drop-down or increase based on the city lockdown precautions in Wuhan. The authors also gave guidance for protection against COVID-19, such as being educated about the virus, social distancing, and lockdown.

In Reference [59], the authors have developed a patient monitoring program that allows daily electronic checking of symptoms, providing advice and reminders via text messages, and providing care by phone. Patients registered in the system complete a daily questionnaire to evaluate 10 symptoms using a scale from 0 to 4. In addition to determining how much they feel the infection is affecting them, the number of analgesic/antipyretic tablets they take, and the temperature measured, questionnaire responses are used to classify patients and specify the care needed. The study focused on three measures, namely the number of patients monitored over time, the daily symptoms score, and daily ED referrals.

Likewise, the authors in Reference [60] developed a mobile app to track the spread of COVID-19 symptoms in the UK by analyzing a set of data reported by patients registered in the app, including location, age, health risk factors, symptoms, healthcare visits, and COVID-19 test results. Survey data helped in determining patients’ type and intensity, availability of personal protective equipment, and work-related stress and anxiety.

The study presented in Reference [61] was concerned with evaluating one of the COVID-19 applications in terms of user satisfaction and the possibility of using the data collected to support decision-makers and healthcare providers. The app collects information daily from patients, including symptoms, vital signs, and an assessment of their satisfaction with the services provided by the app. The data collected is distributed on an interactive map according to the postal code for each user, which helps in knowing the regional distribution of the spread of infection in addition to the percentage of healthcare consumption in each region.

Another study [62] provided an analytical model for predicting patient census and estimating ventilator needs for a given hospital during the COVID-19 pandemic. Through this study, it was noticed that the estimation of the bed and ventilator needs is influenced by the length of hospital stay, and the number of days of inpatient ventilator use. Also, there was no relationship between the age of hospitalized patients and the likelihood of needing a ventilator, or between the inpatient gender and the length of stay. They recommended that each hospital relies on its internal data for accurate resource planning.

Furthermore, the Institute for Health Metrics and Evaluation (IHME) COVID-19 health service utilization forecasting team conducted a study to predict the expected daily use of health services and the number of deaths due to COVID-19 for the next four months from the date of the study for each state in the US [63].

The authors in Reference [64] tried to describe the clinical characteristics and identified factors that predict intensive care unit (ICU) admission for COVID-19 patients. They found that the need for a COVID-19 patient to enter the ICU can be predicted by checking a set of medical parameters that can be easily obtained: age, fever, and tachypnea with/without respiratory crackles. They used the EHRead [65] technique that was developed by Savana to extract information from the medical records. Also, deep learning convolutional neural network classification methods are used to classify the extracted data.

The authors in Reference [66] provided a data-driven framework to pre-assess the risks of the COVID-19 pandemic and to identify high-risk areas in Italy. The framework assesses the risk index using a function consisting of three criteria, namely disease risk, area exposure, and the vulnerability of its population. The twenty Italian regions are classified based on available historical data, which include population density, age, human mobility, air pollution, and winter temperature. The study showed a correlation between the risk index and the number of deaths, infected, and patients in ICU. They also provided a policy model to assist authorities in making several decisions.

Moreover, regional healthcare models have been developed to estimate the pandemic, like the simulation approach developed at the University of Pennsylvania called Monte-Carlo [67]. Such models can be used to manage facilities and plan for an anticipated increase in patient numbers, but not for an estimate of daily operational needs. Applying the Pennsylvania model in an individual hospital requires unknown parameters like the proportion of the region’s patients expected to visit that hospital, and the percentage of the regional population isolated sufficiently to avoid infection.

## 3. Big Data Analytics Tools

Enterprise systems that have functions and functionality for big data applications are known as big data analytics platforms. It helps companies to reveal previously overlooked correlations, market trends, and valuable information from a large amount of big data. Table 3 and Table 4 show the most popular big data analytics platforms and data storage management, respectively.

## 4. Findings, Challenges, and Future Directions

This section is organized as follows. First, Section 4.1 provides our findings from the literature review conducted in Section 2. Section 4.2 discusses the key challenges that were faced when designing big data analytics solutions to address the COVID-19 pandemic. Section 4.3 presents several future directions to be considered by researchers and authorities.

### 4.1. Findings

This section is organized as follows. First, Section 4.1.1 introduces the type and source of data that can be used in healthcare solutions. Then, Section 4.1.2 introduces the type and source of COVID-19 data found in the literature.

#### 4.1.1. Data Type and Source

**Figure 2:**
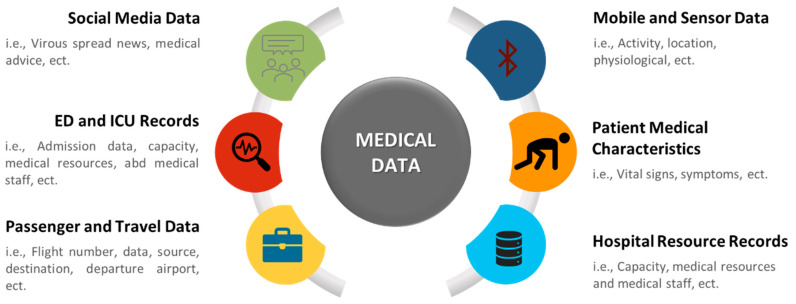
Type and source of medical data.

Numerous data can be utilized in the medical health sector. As shown in Figure 2, medical data can be classified into six categories based on their type and source. Analyzing this data will assist in predicting future events, understanding the current situation, and making several decisions. The medical data can be obtained from many sources, as it can be collected using sensors of wearable/mobile devices or medical devices [39,42,46,53], online questionnaires [55,59], websites or mobile apps [40,41,43,45,60,61], hospital records [50,51,52,62,64], local and international health systems [44,47,57,63,67], interviews and case study samples [54], and data on open databases or social media websites [58].

#### 4.1.2. Data Used in COVID-19 Solutions

**Figure 3:**
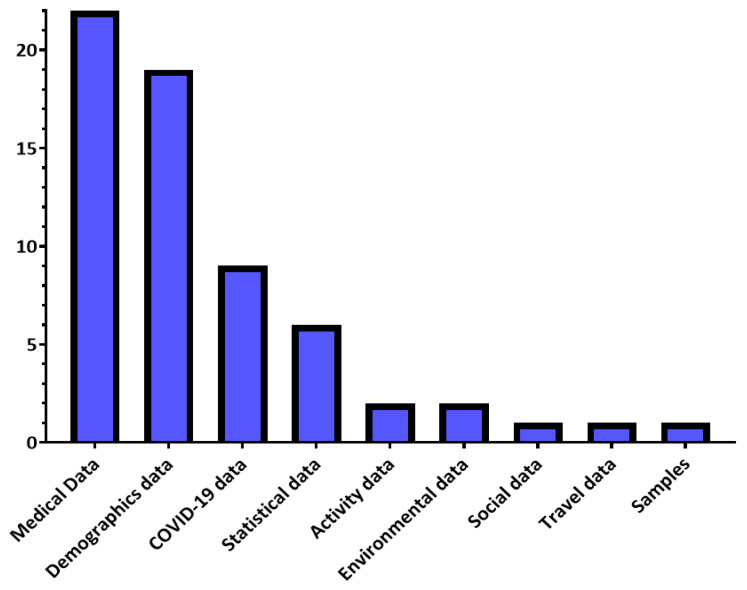
COVID-19 data distribution in the reviewed studies.

Many solutions have been designed to control the COVID-19 pandemic, including diagnosis, forecasting, and decision-making solutions. These solutions use many types of data, shown in Figure 3, which we will introduce in this section based on the survey conducted in Section 2.

Demographic data is useful in understanding the main characteristics of the population and can be used to classify study samples into several categories, such as males and females, to simplify the study of the sample. Social data is also used by solutions that study the impact of the repercussions of the COVID-19 pandemic on the human psychological state. Moreover, there are researchers who have been interested in investigating the possibility of benefiting from activity data and other indicators collected via smartwatches and wearables. Travel data is used to identify suspected COVID-19 cases that have come from countries where the pandemic has spread. Table 5 shows examples of each type of data discussed in this paragraph.

Medical data is widely used in studies directed to control COVID-19, through which it is possible to determine the features of the disease that help in its diagnosis as well as prediction of its occurrence. Additional data on COVID-19 is also used, which helps to know the number, status of cases, and the results of the PCR COVID-19 test. Another type of data relies on sampling to detect virus incubators and contaminated places. Also, statistical data is used for resource management and risk prediction purposes, such as full utilization of ICU capacity, to devise proactive solutions. Finally, the environmental data, which some studies have been interested in, assesses the risks of the spread of the pandemic and determines the areas in which the population will be more vulnerable to infection. Table 6 shows examples of each type of data discussed in this paragraph.

**Figure 4:**
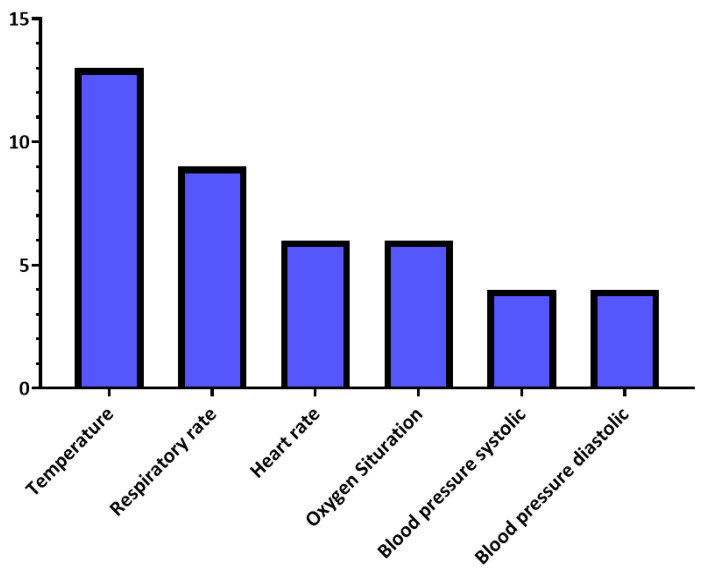
Vital signs’ distribution in the reviewed studies.

**Figure 5:**
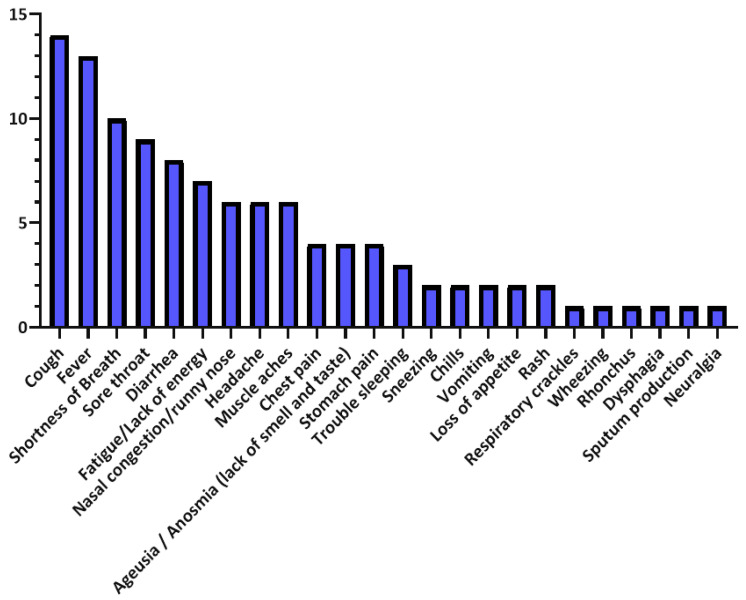
Symptoms’ distribution in the reviewed studies.

Moreover, Table 7 summarizes the vital signs and outwardly measurable symptoms considered by the reviewed studies, where the distributions of vital signs and symptoms in the reviewed studies are presented in Figure 4 and Figure 5, respectively.

Several techniques shown in Table are used to analyze the data presented in this section. However, many other techniques have been used in healthcare, summarized in References [24,71], whereas numerous other applications can be found in the literature. Based on the survey conducted in this paper, the main challenges of applying data analysis techniques when developing solutions to assist in coping with the COVID-19 pandemic are the volume and variety of the data. For example, prediction models developed based on data from a particular hospital may not provide the same accuracy when applied to data from a different source. Therefore, sharing data on the local and international level will serve in improving the accuracy of data analysis solutions.

Furthermore, we found that several tools are used to implement models proposed in the reviewed studies, including R language [43,44,62], R language with Python [42,67], MATLAB [40,57,66], MS Excel [54,62], IBM SPSS [47,54,61], and GraphPad Prism [50].

### 4.2. Key Challenges

Several challenges may hinder the beneficial outcome from the application of big data analysis tools in the health sector that have been encountered when designing solutions to address the COVID-19 epidemic, which will be discussed in the following subsections.

#### 4.2.1. Security and Privacy

Healthcare data security and patient privacy issues [22,72,73,74] are a concern of authorities and even patients, and medical data is only shared under certain conditions and for specific specialists/researchers and purposes. Therefore, it is necessary to define the mechanisms, strategies, and regulations that govern and facilitate access to medical data without compromising patients’ privacy or exploiting the data for unacceptable purposes, especially when critical conditions occur and with the spread of dangerous epidemics that need quick solutions, such as COVID-19.

#### 4.2.2. Sharing Data

Variety and volume of data play a vital role in extracting useful information as well as in understanding various events when applying data analysis tools [51]. For example, the spread of COVID-19 in the city of Wuhan in China raised concerns in other countries about the characteristics of the virus, its impact, as well as determining the countries affected by the epidemic and whether it has been visited by travelers to take preventive measures that limit the spread of infection. This challenge can be overcome by making use of Blockchain technology [75], which helps in large-scale sharing of information securely by anonymizing patients as well as the verified data.

#### 4.2.3. Information Correctness

Although the Internet and social media have a great role in transmitting information and facilitating communication, they are one of the main sources for transmitting false medical information and rumors, for example, about disease, the effects of the virus, and the impact of the vaccine, all of which will hinder the efforts of government and health agencies to contain the spread of virus and the preservation of human health. It may also have negative psychological effects on society. Moreover, the absence or incorrectness of some study data may lead to biased study findings [44]. However, artificial intelligence and big data analytics tools can be used to check and filter information on the Internet and alert people on misinformation and remove it from the network [76].

#### 4.2.4. Patient Cooperation

The patient is the main source for understanding the nature and characteristics of new diseases. Therefore, there is an urgent need to share part of his health information, for example, his medical history record, with the research organizations. Moreover, sharing activity and physiological information gathered from wearables can also contribute to building predictive systems. However, many people are not willing to share their health information with others, as well as other personal information like gender and location [45]. For example, during a survey conducted in January 2020 [14], only 37% of 4600 individuals, without the severe disease, indicated a willingness to blindly share their health information with healthcare research organizations. Therefore, people must be educated about the importance of blind data sharing. Also, to increase people’s confidence in terms of their data privacy, the parties authorized to collect data must be identified as well as the regulations that they adhere to.

### 4.3. Future Directions

Most countries have made many efforts to contain the spread of COVID-19 and mitigate its repercussions, as they have faced various challenges, including the cost and limited capacity for the COVID-19 test. For instance, the Kingdom of Saudi Arabia signed a contract worth 995 million SR with China for 9 million Coronavirus test kits, to perform diagnostics with a capacity of 10,000 tests per day [77]. Another challenge is the lack of a mechanism to monitor the health status of individuals, especially for those who are isolated in their homes. In the United Kingdom, this challenge has resulted in the death of a number of people alone in their homes due to the coronavirus, as their death was not discovered for up to two weeks [78]. Moreover, there is a lack of immediate data to proactively manage resources, such as the distribution of medical staff between regions, as well as the estimated ventilators required for each hospital, which depends on the expected numbers of patients and their different needs. Therefore, we recommend using big data analytics tools to assist stakeholders to make decisions and predict the future. The following are several areas of big data analytics tools’ use that are provided based on the stakeholder level.

#### 4.3.1. Government Level

Social media big data analysis can help spot misinformation about diseases, alert people, and prevent it from spreading. Also, analysis of international air travel data will help track the spread of the pandemic between countries to take proactive preventive measures. Moreover, big data science, including advanced machine learning techniques such as deep learning, mathematical and statistical models such as autoregressive integrated moving average (ARIMA), optimization techniques such as particle swarm optimization (PSO), and simulation models such as SEIR (Susceptible, Exposed, Infected, and Recovered states), can be used to accurately predict the development of the outbreaks like COVID-19. Such models help in forecasting, controlling epidemics, and measuring the impact of interventions and control measures taken by authorities or even planned to be taken. With the available data on COVID-19, these models can be utilized to describe the dynamic aspects of the outbreak to predict early and thus prepare the healthcare infrastructure to manage the impact of such pandemics.

The use of social media has increased during this pandemic. Social media platforms serve as an easy tool for the individual for sharing their views and perceptions. Furthermore, it can also be utilized to get up-to-date information about the pandemic. These colossal amounts of data can be utilized by the government to track the people’s views about the policies and awareness about COVID-19. Several Natural Language Processing (NLP) and AI techniques can be used to track the individual perceptions about the precautionary measures taken by the government. Similarly, some precautionary measures like lockdown, social distancing, remote work, and online education, have isolated the people and, in some cases, may result in some psychological health issues. Several sentiment analysis and opinion mining techniques can enable to pre-emptively detect and diagnose depression levels in the individual. Similarly, these techniques can also be utilized to track the fake news and rumors related to the COVID-19 pandemic.

#### 4.3.2. MoH Level

Analyzing big patient data helps in making proactive resource management decisions, such as the medical staff distribution mechanism and estimating the need for ventilators, as this depends on the expected requirements of patients and their numbers in each city. Big data models such as machine learning help to identify new disease patterns, symptoms, and disease course, as well as allow risk factors associated with the disease. This helps in developing strategies and proactive measures as well as making decisions related to the allocation of medical resources.

Moreover, most smartwatches and wearable devices can measure most of the vital signs shown in Table 7, and collecting and analyzing such data has many benefits, including the following:

1. Large-scale data analysis of the general population and hospital patients would assist the MoH in identifying current health trends among the population and aid in the early prediction of emergencies and epidemics.
2. The monitoring of the vital signs of the general population can say a lot about their health and help in the gauging of stress levels and the overall health of different age groups, particularly the older population. This would help in the establishment of health drives and clinics raising awareness of the appropriate conditions among the population.
3. Analyzing respiratory rate and oxygen saturation data would help in the identification of respiratory problems among the population, including pollution-related respiratory problems among different cities, age groups, and genders.
4. The monitoring of various symptoms on a large scale will also help the MoH gauge in advance the health of the population in general and enable them to take proactive decisions.
5. Centralized real-time data visualization for the number of active and infected cases can help the MoH to identify the areas that contain huge numbers of COVID-19 patients. Furthermore, it can aid the health professionals and decision makers to provide more health facilities in the areas with huge numbers of COVID-19 patients. Similarly, the policy makers can impose strict precautionary measures, and this will reduce the risk of contamination. Big data analysis tools can provide very powerful data analysis and visualization techniques.

#### 4.3.3. Hospital Level

The analysis of remote patient monitoring data can assist in estimating the number of patients in a specific area to optimally plan for containing any expected increase in the number of patients beyond the hospital capacity. Moreover, health data is growing exponentially, making it difficult to use traditional representation methods such as tables. The employment of artificial intelligence alongside data analytics tools has a role in addressing this challenge, and it can help in the extraction and representation of data in real-time—the Savana system [65] is an example.

Implication of AI and ML techniques in automated early diagnosis and prognosis of several diseases in general and COVID-19 specifically has shown the significant outcomes. Similarly, a remote COVID-19 patients triaging system allows to remotely monitor the patients. The emergence of non-invasive medical devices and the integration of sensors in smart devices and watches facilitate the process of remote monitoring. The data generated by the sensors will be utilized by AI and ML algorithms for diagnosis and prognosis. Due to the huge number of COVID-19 patients and the risk of contamination, these applications will allow the patients with the mild condition to be monitored remotely by the doctors.

#### 4.3.4. Individuals/Patients Level

Real-time analysis of hospital data that are related to admitted patients, waiting lists, and hospital capacity helps individuals locate less crowded hospitals with earlier appointments and less waiting time. Also, linking patient data to maps can help identify areas of infection and provide warnings to people when they are in these areas to reduce the chance of infection. Moreover, the employment of advanced machine learning models such as deep learning can help classify many respiratory diseases by applying them to large samples of coughing and breathing sounds. Integrating such models into mobile apps helps provide a rapid mechanism for individuals to pre-diagnose respiratory symptoms and determine the need for diagnosis by clinicians.

#### 4.3.5. Responsible Authorities Level

Analyzing mobile data helps in identifying polluted public places for disinfection and quarantining infected people and their contacts, even if they do not show any symptoms. Moreover, the integration of mathematical models of the spread of infectious diseases with interactive maps and GPS technology can help in determining the locations and paths of infected people, which allows the imposition of a quarantine only on infected people and not others. In turn, this will reduce the economic damages caused by suspending all activities during the quarantine period for all people.

## 5. Conclusions

The volume of data increases dramatically over time, especially data generated on the global pandemic caused by COVID-19. Such volume of data requires utilizing big data analytics tools along with AI techniques to make sense of the pandemic and control its spread in a timely manner. In this study, we presented a review of several data analysis applications for COVID-19, providing a taxonomy structure which classified the potential applications of COVID-19 into four categories, namely diagnosis, estimate or predict risk score, healthcare decision-making, and pharmaceutical. The paper introduced several data analysis tools and explained the main features of each tool. We also provided important insights on a number of challenges that might hinder the use of data analytics tools for COVID-19. These challenges include healthcare data security and patient privacy issues, the difficulty of sharing data with researchers, absence of data validation for some studies that may lead to biased results, and the patients’ cooperation in sharing part of their medical information. Finally, we highlighted and discussed a number of future directions that should be considered in further research and applications to assist stakeholders, such as governments, MoHs, hospitals, patients, and responsible authorities, to make decisions and predict the future.

## Figures and Tables

**Table 1:** Summary of surveys on big data analytics in the healthcare field.

Source	Publication Year	Domain	Key Contribution
[22]	2017	Healthcare security and privacy	Discussed healthcare data security and privacy issues, and the mechanisms and strategies available for healthcare data privacy, security, and user access
[23]	2017	Heart attack prediction and prevention	Identified the uses and technologies of big data analytics in this area, as well as challenges and concerns regarding patient privacy
[24]	2018	General healthcare	Defined the scope of big data analytics and its applications in healthcare, and provided strategies to overcome its challenges
[25]	2019	Health care organizational decision-making	Identified the main characteristics and drivers of market uptake of Artificial Neural Networks (ANN) for healthcare-related regulatory decision-making
[26]	2019	Healthcare and medical problems	Reviewed traditional and fuzzy decision-making methods applied to nine areas of healthcare and medical problems
[27]	2019	Healthcare sector applications	Discussed the impact of big data on various stakeholders and the challenges
[28]	2019	IoT and healthcare industry	Identified research trends of the Internet of Things Big Data Analytics model (IoTBDA) in the healthcare industry, and demonstrated the influence of the IoTBDA model on the design, development, and application of IoT-based innovations in healthcare services
[29]	2019	Medical decision-making	Described the current state of research related to collective intelligence
[30]	2019	Patient-centric healthcare system	Presented several analytical approaches from various stakeholders’ perspectives and reviewed the different big data frameworks in terms of data sources, analytical capability, and application areas. Also, it discussed the impact of big data on improving the healthcare ecosystem
[31]	2019	Public health and healthcare organizations	Provided a better understanding for governments and health policymakers about how developing a data-driven strategy could improve public health and the functioning of healthcare organizations and explain the challenges associated with this improvement
[19]	2020	COVID-19 detection and contact tracing	Explained the potentials of nature-inspired computing (NIC) models for accurate COVID-19 detection and optimized contact tracing
[32]	2020	COVID-19 medical images	Discussed the role of medical imaging integrated with artificial intelligence (AI) in combating COVID-19
[33]	2020	COVID-19 medical images detection and classification in terms of evaluation and benchmarking	Highlighted the gaps and challenges, and proposed a detailed methodology for the benchmarking and evaluation of AI techniques used in all COVID-19 medical images classification tasks
[21]	2020	COVID-19 pandemic	Explained the role of AI in fighting pandemics
[34]	2020	Data harmonization (DH) and health management decision-making	Collected definitions and concepts of DH and addressed the causal relation between DH and decision-making in health management
[35]	2020	Healthcare aspects	Provided an overview of the big data analytics publication dynamics in healthcare and discussed several examples to this field
[36]	2020	Healthcare engineering systems	Synthesized and analyzed publications covering data analytics, big data, data mining, and machine learning in the field of Healthcare Engineering Systems
[37]	2020	Mobile health (m-health)	Explored AI applications and big data analytics to provide insights for users to plan resource use for specific challenges in m-health, and proposed a m-health model based on AI and big data analytics

**Table 2:** Data analysis technique, type, source, and findings of the existing studies.

Area	Ref	Aim	Technique	Used Data Type	Data Source	Findings
Diagnosis	[39]	Develop a diagnosis model for COVID-19 detection and diagnosis of symptoms to define appropriate care measures	Best Worst Method (BWM)	Symptoms and CT scans	Body sensors	The model can differentiate COVID-19 from four other viral chest diseases with 98% accuracy
	[40]	Design a medical device to detect and track respiratory symptoms of COVID-19	N/A	Symptoms	Headsets and mobile phone	The approach provided good and stable results and can be expanded to include more sensors to detect other COVID-19 symptoms
	[41]	Develop a remote patient monitoring program (RPM) for discharged COVID-19 cases	The mixed-effects logistic regression model	Demographics, medical data	The remote monitoring program, pulse oximeter, and thermometer	RPM provides scalable remote monitoring capabilities and decreases readmission risk
	[42]	Investigate smartwatches usefulness in pre-symptoms COVID-19 detection	Two anomaly detection models (RHR-Diff and HROS-AD)	Demographics, activity, medical data, COVID-19 status	Smartwatches and MyPHD mobile app	Respiratory infections can be detected through activity tracking and health monitoring via wearable devices
	[43]	Identify symptoms associated with positive COVID-19 cases	Principal component analysis (PCA), and logistic regression model	Demographics, medical data	Screening via phone and COVID-19 PCR test	Fever, anosmia/ageusia, and myalgia were the strongest signs of positive COVID-19 cases, while no symptoms were limited to nasal congestion/sore throat associated with negative cases
	[44]	Determine the clinical characteristics and outcomes of COVID-19 patients in the NY area	N/A	Demographics, medical data, COVID-19 status	Northwell Health system	The common comorbidities were obesity, hypertension, and diabetes.From outpatients or dead patients (*n* = 2634): 21% died, 14.2% were treated in the ICU, 12.2% received MV, and 3.2% were treated with kidney replacement
	[45]	Distinguish COVID-19 cough sound from other respiratory diseases through crowd source data	Logistic Regression (LR), Gradient Boosting Trees, and Support Vector Machines (SVMs)	Demographics, medical data, COVID-19 data	Web app and Android app	Wet and dry cough are the common symptoms of positive COVID-19 cases, whereas chest tightness and the lack of smell are the common combination symptoms
	[46]	Discuss the importance of developing complementary technologies to diagnose and monitor COVID-19 infections	N/A	Activity data, medical data	Sensors	Recommend deploying advanced wearable technologies configured to directly address needs in COVID-19 monitoring and noticing the symptoms
	[47]	Identify the clinical characteristics of COVID-19 to help in mapping the disease and guiding pandemic management	N/A	Demographics, medical data, COVID-19 status, travel data	Health Electronic Surveillance Network (HESN) database for all Saudi Arabia regions	Fever and cough were common symptoms in the study sample
	[48]	Employing a two-stage cascading platform to enhance the accuracy of machine learning models	Progressive machine learning technique merged with Spark-based linear models, Multilayer Perceptron (MLP), and LSTM	Medical data	Cardiac Arrhythmia Database. Uniform Resource Locator (URL) Reputation Dataset from University of California Irvine Machine Learning (UCI ML) Repository	Using an improved algorithm with two-step data analysis platforms can increase accuracy in lower computation time
	[49]	Analyzing the dense layers among the convolutional network can help to increase the accuracy of classification of images for diabetic retinopathy	Deep learning model	Medical data, Demographics data	The Messidor-2 dataset from the hospital	Using improved programming technology can enhance accuracy
	[50]	Analyze the effects of COVID-19 on patients with cardiovascular disease	Generalized linear mixed model	Demographics, medical data, COVID-19 status	HERs from General Hospital of Central Theatre Command in Wuhan, China	Middle-aged and elderly heart patients are most likely to have COVID-19, whereas new-onset hypertension and heart injury are common complications of severe COVID-19 cases
Estimate or Predict Risk Score	[51]	Specify the effect of COVID-19 on the cardiovascular system	The multi -factor logistic regression model	Demographics and medical data	HERs	Cardiac function and vital signs should be monitored in COVID-19 patients, especially those with hypotension, pericardial effusion, or severe myocardial injury
	[52]	Develop and validate a risk score to predict adverse events of suspected COVID-19 patients	Least absolute shrinkage and selection operator (LASSO) and logistic regression models	Demographics and medical data	15 EDs in Southern California	COVAS score can help physicians to identify patients who may experience a serious event within 7 days
	[53]	Discover unregistered suspected COVID-19 patients and infectious places	SIR and θ-SEIHRDmathematical models	Demographics and COVID-19 data	IoT-based system and GPS	The proposed system helps identify people who had close contact with COVID-19 patients
	[54]	Verify if the COVID-19 virus can be transmitted through indirect contact	N/A	Demographics, medical, environmental, and other data	Guangzhou CDC database and sample collection	The virus can survive for a short period on surfaces, allowing indirect transmission of infection to uninfected people
	[55]	Identify the COVID-19 outbreak impact on the psychological side	Bivariate linear regression	Demographics, medical, social data	Online questionnaire	The COVID-19 outbreak has a significant mental impact on people
	[56]	Analyze the risk of tuberculosis skin on getting infected by tuberculosis	Statistical	Medical data, Demographics data	Public source	The tuberculin skin can increase the infection by up to 20%
	[57]	Predict the course of the COVID-19 epidemic to design a control strategy	A designed mathematical model called SIDARTHE	Demographics, medical, environmental data	Public data from Italian MoH and Italian Civil Protection	Social distancing measures and lockdowns are necessary and effective, and precautionary measures for COVID-19 can only be relieved when tests are conducted on a large scale and a mechanism for contact tracing is in place
Healthcare Decision-Making	[58]	Evaluate the effectiveness of COVID-19 control measures	C-SEIR model(mathematical model of disease transmission dynamics)	Confirmed COVID-19 data	Public data sources	Quarantine measures have an effective role in containing COVID-19, but they are economically expensive
	[59]	Develop a patient monitoring platform to directly provide the necessary care	N/A	Demographics, medical, COVID-19 data	Online questionnaire via patient monitoring program	Analyzing patient monitoring data helps to know the risk score to determine the care required, allowing optimal consumption of medical resources
	[60]	Provide a platform for data collection and analysis to estimate disease incidence to develop risk mitigation strategies and resource allocation	Weighted prediction model	Demographics, medical, COVID-19, and other data	Mobile app	Existing data collection methods can be repurposed to track and obtain real-time data for the population during any rapid global health crisis
	[61]	Identify the regional distribution of the spread of infection and the percentage of healthcare consumption in each region	N/A	Demographics, medical, and other data	Mobile app	Can rely on the mobile app to perform self-assessment and data collection that can be displayed on an interactive map and linked to the results of the COVID-19 test results to support decision-makers and healthcare providers in making decisions
	[62]	Forecast the census and ventilators requirements for a specific hospital	Weibull and conditional distributions (analytical model)	Statistical data	COVID-19 hospitalized patient records	The model can predict the census and the required number of MV in one, three, and seven days after the simulation run date
	[63]	Estimate the need for health services and the number of daily deaths over the next 4 months from the date of the study	Statistical model	COVID-19 and other data	WHO websites and local and national authorities in the US states	The model predicts an increased death rate and demand for medical beds, ICU, and MVs
	[64]	Prove that the three clinical variables: age, fever, tachypnea, can be used to predict the need to admit COVID-19 patients into the ICU	EHRead from Savana [65], and deep learning convolutional neural network classification methods (Prediction model)	Demographics, medical data	EHRs of the hospitals within the Servicio de Salud de Castilla-La Mancha (SESCAM) Healthcare Network in Castilla-La Mancha, Spain	The most common symptoms of male COVID-19 with an average age of 58.2 years who were admitted to ICU are coughing, fever, and shortness of breath, while those between 40 and 79 years of age are likely to be admitted to the ICU if they suffer from rapid breathing
	[66]	Pre-risk assessment of the epidemic in Italy and identification of high-risk areas	a-priori effect of hazard and vulnerability model (a-priori E_H_V)	Statistical and environmental data	Data from Italian Ministry of Economic Policy Planning and Coordination, Italian Ministry of Health website, WHO, Italian Ministry of Agriculture, and ISTAT database	The risk of a pandemic is higher in some northern regions of Italy and the policy model developed can help policymakers make decisions
	[67]	Estimate the remaining period before consuming the operational capacity of the hospital and its resources	Monte Carlo simulation, SIR model, and COVID-19 Hospital Impact Model (CHIME)	Statistical data	Academic health system for three hospitals in the Philadelphia region	The model can help in making proactive decisions

Note: CT: chest computed tomography, CDC: center for disease control and prevention, COVAS: COVID-19 acuity score, CHIME: COVID-19 hospital impact model, C-SEIR: conscious-based susceptible exposed infected recovery, ED: emergency department, HERs: electronic health records, HROS-AD: heart rate over steps anomaly detection, ISTAT: Italian National Institute of Statistics, GPS: global positioning system, ICU: intensive care unit, LSTM: long short-term memory, IoT: internet of things, MoH: Ministry of health, MV: mechanical ventilation, N/A: not available, NY: New York, RHR-Diff: resting heart rate difference, SIDARTHE: susceptible (S), infected (I), diagnosed (D), ailing (A), recognized (R), threatened (T), healed (H) and extinct (E), SIR: susceptible-infected-recovered, θ-SEIHRD: susceptible exposed infectious hospitalized recovered dead, θ: is the fraction of detected infected people, US: United State, WHO: world health organization.

**Table 3:** Most popular big data analytics tools.

Tool	Description	Main Features	Availability	Reference
Apache Hadoop [68]	Data storage and distributed processing.	Distributed parallel processing of large amounts of data by using Hadoop Distributed File System (HDFS), and the MapReduce YARN (“Yet Another Resource Negotiator”)	Open source	https://hadoop.apache.org/, Accessed on: 18 March 2021
IBM [69,70]	IBM provides a variety of big data tools including: IBM big SQL Apache Spark Big Integrate	Text Analytics Data Visualize Artificial Intelligence	Commercial	https://www.ibm.com/analytics/hadoop/big-data-analytics, Accessed on: 18 March 2021
Amazon [68]	Data analysis systems	Data Storage Data Analytics	Commercial	https://aws.amazon.com/products/, Accessed on: 18 March 2021
Microsoft Azure [68]	It is a big data platform that is cloud-based and used for developing, analyzing, installing, and managing applications.	It provides the following services: Software as a service (SAAS). Platform as a service, Infrastructure as a service.	Azure free account and get popular services free for 12 months.	https://azure.microsoft.com/en-us/, Accessed on: 18 March 2021
Qubole	It is an easy, open, and stable Data Lake Platform for machine learning, streaming, and ad-hoc analytics.	Platform that drives an ETL (extraction, transformation, and load): Machine Learning AD-HOC Analytics	Commercial	https://www.qubole.com/, Accessed on: 18 March 2021
HPCC	Tool that offers a framework for data processing with a single architecture.	Data integration and cluster management are easy. Using the ETL engine and the ECL scripting language, data is extracted, transformed, and loaded.	Open source	https://hpccsystems.com/, Accessed on: 18 March 2021
MapR	MapR supports all Hadoop APIs and Network File System (NFS).	Hadoop, Spark, and Apache Drill MapR supports all Hadoop APIs and Network File System (NFS).	Open source	https://www.hpe.com/us/en/software/data-fabric.html, Accessed on: 18 March 2021
KNIME	Data Mining New futures prediction.	Build and visual workflows. Machine learning advanced predictive Interactive data views and reporting	Open Source	https://www.knime.com/knime-analytics-platform, Accessed on: 18 March 2021
Datameer	Integrate data with different engines. Built on the top of Hadoop.	Datameer Spotlight combines virtual data management and easy modeling tools. Datameer Spectrum is a robust, non-coding ETL++ tool and platform	Commercial	https://www.datameer.com/, Accessed on: 18 March 2021

**Table 4:** Data storage and management.

Data Storage	Description	Website
Cloudera	It extends the Hadoop with extra services	https://www.cloudera.com, Accessed on: 18 March 2021
Apache Cassandra	Distributed database management system, multiple servers	https://cassandra.apache.org/, Accessed on: 18 March 2021
Chukwa	Hadoop distributed file system (HDFS)	http://chukwa.apache.org/, Accessed on: 18 March 2021
Apache HBase	Hadoop distributed file system (HDFS)	http://hbase.apache.org/, Accessed on: 18 March 2021
MongoDB	Document-oriented database	https://www.mongodb.com/, Accessed on: 18 March 2021
Neo4j	java—graph database	https://neo4j.com/, Accessed on: 18 March 2021
CouchDB	Globally distributed server-clusters	https://couchdb.apache.org/, Accessed on: 18 March 2021
Terrastore	Distributed Database Management System (DBMS) that provides per-document consistency guarantees	https://code.google.com/archive/p/terrastore/, Accessed on: 18 March 2021
HibariDB	Hibari is a distributed, ordered key-value store	https://hibari.readthedocs.io/en/latest/index.html, Accessed on: 18 March 2021
Riak	NoSQL database, cloud storage	https://riak.com/, Accessed on: 18 March 2021

**Table 5:** Demographics, social, activity, and travel data found in the reviewed studies.

Data Category	Data Type	Studies
Demographics data	Gender	[41,42,43,44,45,47,50,51,52,55,59,60,61,64]
	Age	[41,42,43,44,45,47,49,50,51,52,55,56,57,59,60,61,64]
	Height	[42,60]
	Weight	[42,60]
	Body mass index (BMI)	[52]
	Language	[41]
	Race	[41,44,52,59,60]
	Ethnicity	[41,42,44,59,60]
	Nationality	[47]
	Religion	[55]
	Marital status	[55]
	Median income	[41]
	Zip code/postal code	[41,60,61]
	Location/geolocation	[45,53,60]
	Region	[47]
	Insurance	[44]
	Job/educational institute	[47,54,55]
	Number of family members	[55]
Social data	Social stressors	[55]
Activity data	Steps	[42]
	Sleep	[42]
	Heart rate	[42]
	Home-quarantine activities	[55]
Travel Data	Recent outside travel history	[47]
	Outside destinations	[47]

**Table 6:** Medical, COVID-19, samples, statistical, and environmental data found in the reviewed studies.

Data Category	Data Type	Studies
Medical data	Vital signs	[41,42,43,44,46,47,50,51,52,54,55,57,59,60,61,64]
	Symptoms	[39,40,41,42,43,45,46,47,48,49,50,51,52,54,55,56,57,59,60,61,64]
	Comorbidities	[42,44,47,50,51,52,60,61,64]
	Medical history	[45,60]
	Routinely taken medications	[42,59]
	Laboratory findings	[47,50,51]
	CT scans	[39,48,50,54]
	Required ICU	[41]
	ICU length of stay	[41]
	Readmission status	[41]
COVID-19 data	Number of cases and status	[42,44,47,50,53,58,63]
	Test date	[42,44]
	Results (laboratory, outcome)	[42,45,47,50,60]
	Symptom onset date	[42]
	Incubation periods	[47]
	Treatment measures	[50]
	Infection feels	[59]
Samples	Throat swabs	[54]
	Blood samples	[54]
	Aerosol and surface samples	[54]
Statistical data	Healthcare visits	[60]
	Hospital capability and utilization	[63,67]
	Known regional injuries	[67]
	Percentages related to ICU	[67]
	Future daily admissions	[62]
	Percentage of inpatients requiring MV	[62]
	ICU lengths of stay	[62]
	Duration of MV	[62]
	App satisfaction assessment	[61]
	Hospital market share	[67]
	Population age and size	[66,67]
Environmental data	Epidemiological data	[57]
	Air pollution	[66]
	Winter temperature	[66]
	Healthcare density	[66]
	Human mobility	[66]
	Housing concentration	[66]

Note: ICU: intensive care unit, MV: mechanical ventilation.

**Table 7:** Summary of vital signs and outwardly measurable symptoms considered by the existing studies.

Data Category	Data Type	Studies
Vital signs	Temperature	[41,42,43,44,47,50,51,52,54,59,60,61,64]
	Heart rate	[44,46,47,50,51,52,54]
	Respiratory rate	[40,44,46,47,50,51,52,54,61,64]
	Blood pressure systolic	[47,50,51,52,54]
	Blood pressure diastolic	[47,50,51,52,54]
	Oxygen saturation	[41,44,47,50,51,52,54]
Symptoms	Fever	[39,43,45,47,50,51,52,54,55,59,60,61,64]
	Shortness of breath	[39,41,43,45,50,59,60,61,64]
	Respiratory crackles	[64]
	Wheezing	[64]
	Rhonchus	[64]
	Chest pain	[50,51,60,64]
	Cough	[39,40,41,43,45,46,47,50,54,55,59,60,61,64]
	Sneezing	[43,61]
	Chills	[39,50]
	Nasal congestion/runny nose	[39,43,47,50,54,55]
	Ageusia/Anosmia (lack of smell and taste)	[43,45,60,64]
	Headache	[39,43,45,47,50,64]
	Sore throat	[39,43,45,47,50,55,59,61,64]
	Dysphagia	[64]
	Sputum production	[39]
	Fatigue/lack of energy	[39,41,50,55,59,60,61]
	Muscle aches	[43,45,50,51,59,64]
	Diarrhea	[43,47,50,55,59,60,61,64]
	Vomiting	[41,50]
	Loss of appetite	[41,50]
	Trouble sleeping	[42,50,59]
	Stomach pain	[43,50,59,60]
	Rash	[43,56]
	Neuralgia	[64]

## Data Availability

Data was obtained from King Fahd Hospital of Imam Abdulrahman Bin Faisal University and are available from the authors with the permission of King Fahd Hospital of the University.
